# Glial cell alterations in diabetes-induced neurodegeneration

**DOI:** 10.1007/s00018-023-05024-y

**Published:** 2024-01-18

**Authors:** María Llorián-Salvador, Sonia Cabeza-Fernández, Jose A. Gomez-Sanchez, Alerie G. de la Fuente

**Affiliations:** 1 Diabetes and Metabolism Research Unit, Vall d’Hebron Research Institute, Universitat Autònoma de Barcelona, Barcelona, Spain; 2 Wellcome-Wolfson Institute for Experimental Medicine, Queen’s University, Belfast, UK; 3 Institute for Health and Biomedical Research of Alicante (ISABIAL), Alicante, Spain; 4 Institute of Neuroscience CSIC-UMH, San Juan de Alicante, Spain

**Keywords:** Diabetes, Neurodegeneration, Glial cells, Diabetic neuropathy, Diabetic retinopathy, Cognitive decline, Muller, Microglia, Astrocytes, Schwann cells

## Abstract

Type 2 diabetes mellitus is a global epidemic that due to its increasing prevalence worldwide will likely become the most common debilitating health condition. Even if diabetes is primarily a metabolic disorder, it is now well established that key aspects of the pathogenesis of diabetes are associated with nervous system alterations, including deleterious chronic inflammation of neural tissues, referred here as neuroinflammation, along with different detrimental glial cell responses to stress conditions and neurodegenerative features. Moreover, diabetes resembles accelerated aging, further increasing the risk of developing age-linked neurodegenerative disorders. As such, the most common and disabling diabetic comorbidities, namely diabetic retinopathy, peripheral neuropathy, and cognitive decline, are intimately associated with neurodegeneration. As described in aging and other neurological disorders, glial cell alterations such as microglial, astrocyte, and Müller cell increased reactivity and dysfunctionality, myelin loss and Schwann cell alterations have been broadly described in diabetes in both human and animal models, where they are key contributors to chronic noxious inflammation of neural tissues within the PNS and CNS. In this review, we aim to describe in-depth the common and unique aspects underlying glial cell changes observed across the three main diabetic complications, with the goal of uncovering shared glial cells alterations and common pathological mechanisms that will enable the discovery of potential targets to limit neuroinflammation and prevent neurodegeneration in all three diabetic complications. Diabetes and its complications are already a public health concern due to its rapidly increasing incidence, and thus its health and economic impact. Hence, understanding the key role that glial cells play in the pathogenesis underlying peripheral neuropathy, retinopathy, and cognitive decline in diabetes will provide us with novel therapeutic approaches to tackle diabetic-associated neurodegeneration.

**Graphical abstract:**

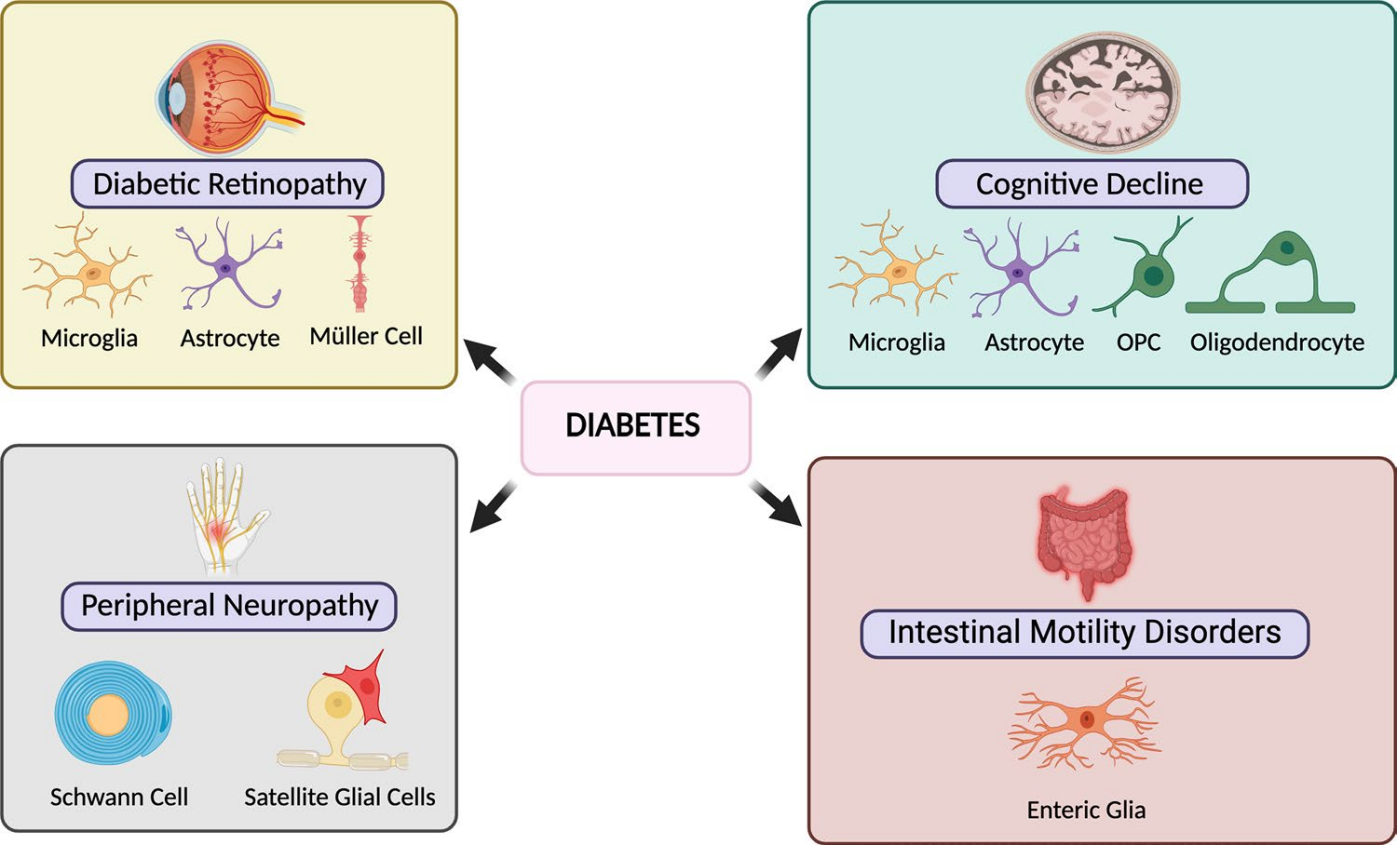

## Background

In an increasingly aging population, the growing prevalence of chronic diseases such as diabetes represents a serious challenge for healthcare systems. Diabetes prevalence is expected to increase to 643 million affected people in 2030, doubling the number of people suffering from diabetes since 2011[1, 2]. This rapid and concerning escalation is predominantly linked to the increased prevalence of type 2 diabetes mellitus (T2D). T2D represents one of the most common metabolic diseases in Western societies and it is considered “the epidemic of the twenty-first century”, affecting 1 in 11 people in Europe [2]. In addition, 318 million adults are estimated to have pre-diabetes or impaired glucose tolerance, representing a population at higher risk of further developing diabetes [2]. Diabetes-associated medical care represents around the 8–12% of the total National Health System expenditures [3–5] due to its strong association with several disabling complications, including alterations in the peripheral (PNS) and central nervous system (CNS) such as peripheral neuropathy, and diabetic retinopathy or cognitive decline, respectively [5–8].

Diabetic retinopathy (DR) is one of the most common diabetic complications and represents one of the leading causes of preventable visual impairment in the working-age population in developed countries, accounting for the 15–17% of all cases of total blindness in Europe and USA [1, 9]. Due to its limiting and debilitating nature, DR severely impacts in the healthcare and social costs of diabetic patients [10]. Current available treatments for DR include laser photocoagulation, intravitreal injections of anti-VEGF agents or corticosteroids. However, all these treatments are invasive and expensive, have a significant number of side effects, and are used exclusively in advanced stages of the disease, when the vascular phenotype becomes evident, and the vision has already been significantly affected. Therefore, new, and more efficient preventive and interventional strategies based on a better understanding of the pathogenesis of the disease are urgently needed.

Diabetic peripheral neuropathy (DPN) is also commonly described as a diabetic complication since 50–66% of diabetic patients will eventually develop DPN during the time course of the disease [11, 12]. DPN is characterized by a progressive distal-to-proximal degeneration of peripheral nerves, which results in sensory symptoms, including spontaneous pain, allodynia (painful sensati5on to innocuous stimuli), hyperalgesia (increased pain perception to noxious stimuli), weakness, and/or paraesthesia and numbness, which vary in nature and severity depending on the specific neuronal subpopulation affected [12]. Although some patients with DPN do not present any symptoms, the vast majority report pain and/or loss of function in distal regions such as in their toes, feet, fingers, or hands. The early onset of DPN is characterized by the hyperexcitability of the sensory nerve fibers, which translates into pain symptoms. Later stages are characterized by a progressive loss of neuronal fibers, and thus significant morbidity and mortality [12]. Similar to neuropathic pain with other etiology, DPN is refractory, partially responsive to existing pharmacotherapy [13] or its adverse effects limit its clinical use.

In addition to DR and DPN, diabetes is strongly associated with cognitive impairment and dementia, with diabetic patients showing reduced performance in multiple cognitive functions. Hence, diabetes and, especially T2D, is often considered an accelerator of cognitive decline (CD) [14]. The tight relationship between diabetes and cognitive impairment and the accumulation of neurological symptoms observed in diabetic patients has driven the growth of epidemiological and clinical studies, including reviews and meta-analysis studies, aiming at establishing a relationship between the two [7, 15]. It has already been established that T2D patients have twofold higher risk of developing Alzheimer’s disease (AD) and vascular dementia, with approximately 30% of T2D diabetic patients over 65 years old showing mild cognitive impairment[16]. Due to this close association and the commonly shared mechanisms between diabetes and Alzheimer’s disease (AD), some researchers have suggested using the terminology “Type -3-Diabetes” for Alzheimer’s disease [17]. CD in diabetic patients is predominantly linked to higher levels of tau and phosphorylated tau in the cerebrospinal fluid, while only 39% of diabetic patients show positivity for Aβ plaques [17, 18]. These features have also been observed in the two most common animal models of type 1 and T2D, Streptozotocin (STZ) and db/db mouse models, respectively [19, 20].

Therefore, diabetes represents a health and economic burden, and thus demands cost-effective and innovative therapeutic strategies to limit its epidemic escalation and the debilitating effects driven by its complications. To do so, determining the underlying mechanisms associated with diabetic complications is of paramount importance.

Despite being primarily a metabolic disorder in which PNS- and CNS-associated complications (such as DR, DPN, and CD-AD) have been linked mainly to microvascular pathology, in the recent years, it has become clear that diabetes significantly impacts both, neuronal and glial cell function in the retina, brain, and the PNS. A growing body of evidence supports the idea that neuronal damage and glial cell alterations are already present in the early stages of DR and DPN, while becoming more dominant in later stages in the CNS [21–23]. Hence, it has been widely hypothesized that neuroprotective therapeutic strategies may be effective in preventing and arresting diabetes-associated neurodegeneration [22, 24]. In the case of DR, retinal functional abnormalities are detectable even before microvascular lesions appear, with especial emphasis been placed in the pathophysiology of the neurovascular unit (NVU), which consists of vascular elements (endothelial cells and pericytes), the basement membrane, neurons, and glial cells (Müller cells, astrocytes, and microglia), which dysfunction greatly impacts neuronal function and may initiate both neuronal degeneration and microvascular impairment [22]. In the PNS, a large amount of data supports the essential role of glial cells, namely satellite glial cells (SGCs) and Schwann cells (SCs) as key components of neuronal structure and function maintenance and therefore, of DPN pathogenesis [25]. On the other hand, diabetic CNS alterations in astrocytes, microglia, and myelin have predominantly been described in association with brain atrophy, hyperpermeability of the blood–brain barrier (BBB) and cognitive decline [26, 27].

**Fig. 1:**
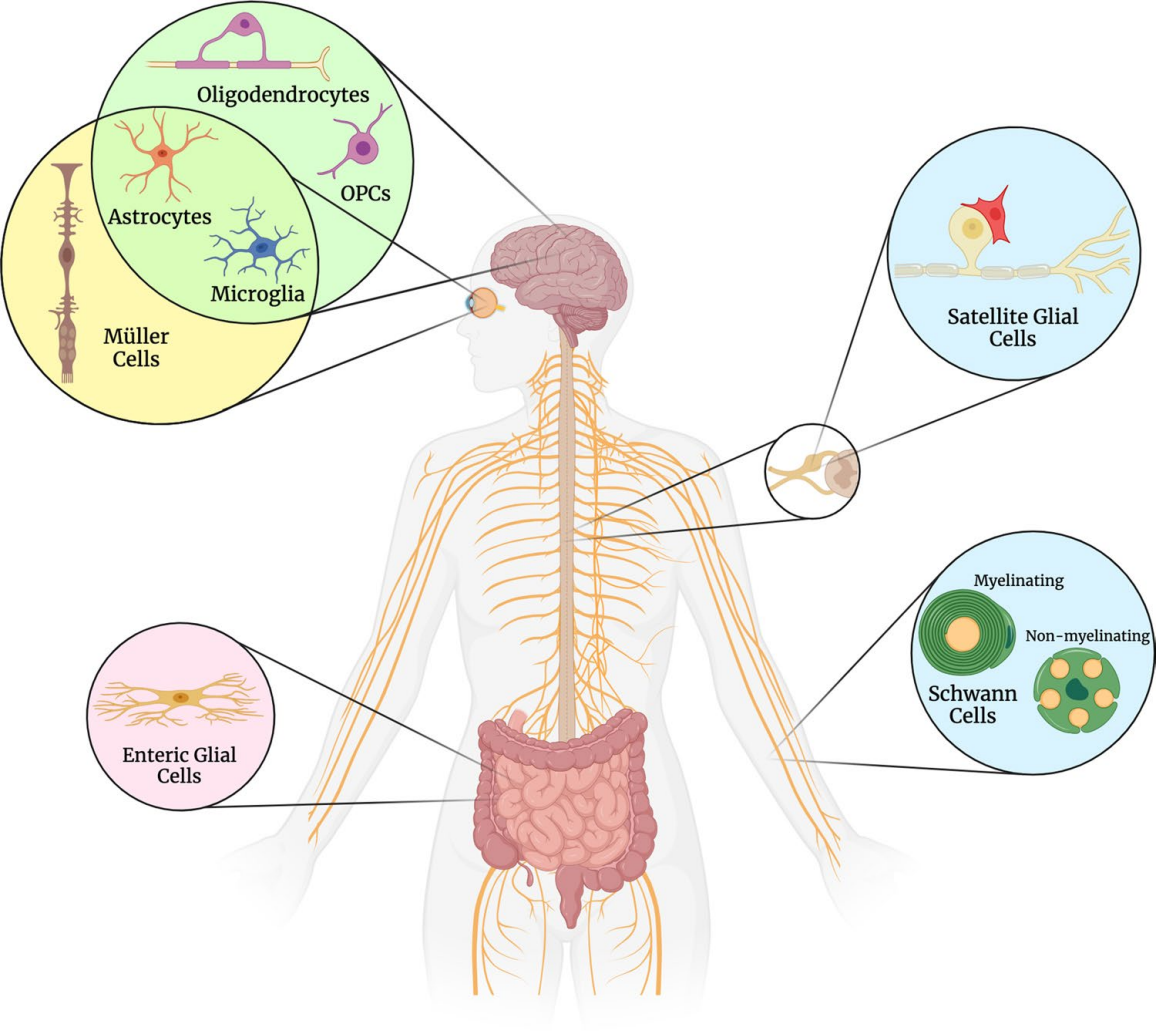
Glial cells across the nervous system are implicated in diabetic complications. Schematic summary of the different central nervous system, peripheral nervous system, and enteric nervous system glial cells which functions are described in this review. This figure was created with BioRender.com

Thus, in this review, we aim to describe common and cell-type-specific alterations observed in the different glial cell populations (Fig. 1) in diabetes to better understand the mechanisms underlying central and peripheral nervous system diabetic complications such as DR, DPN, and CD-AD.

## Central nervous system glia

### Astrocytes and Müller cells

Astrocytes are found in the CNS (spinal cord, brain, and retina) and have a key crucial role in neuronal trophic support, neuronal signal transduction, vessel growth, and BBB and blood–retinal barrier (BRB) maintenance [28, 29]. Müller cells, on the other hand, are the main glial cell and the sentinels of the retina, having a pivotal role in both, retinal homeostasis and in pathological conditions, including DR. Müller cell functions include NVU support, releasing trophic factors, neurotransmitter recycling (e.g., glutamate, γ-aminobutyric acid), controlling water and ion (mainly K^+^) homeostasis, stabilizing microvasculature, regulating retinal innate immunity, and participating in the visual cycle [30]. The role of astrocytes in DR is still uncertain as they show the opposite reaction to Müller cells in hyperglycemia. While Müller cells become gliotic and hypertrophic associated with morphological alterations, the number of astrocytes is significantly decreased already in early DR [31], possibly leading to BRB impairment and vascular leakage. However, although debatable, Müller cells have also been reported to die via caspase-1-mediated pyroptosis in DR[32–34] exacerbating neuroprotection loss, BRB impairment, and vascular leakage [30, 34, 35]. Due to astrocyte and Müller glial cell relevance in maintaining the neurovascular structure and proper CNS function, it is believed that their dysfunction in diabetes may be a crucial factor in the pathophysiology of DR and diabetes-associated cognitive impairment, contributing to both microvascular and neurodegenerative lesions [7, 30].

#### Gliosis and inflammation

Müller cell gliosis has been described as one of the first pathogenic events and a hallmark of DR, being present even in the absence of vascular or clinical symptoms and persisting throughout the disease course. Gliotic Müller cells release a myriad of pro-angiogenic factors, pro-inflammatory, and pro-fibrotic factors that contribute to vascularization and neurodegeneration, and therefore to DR progression [30, 34, 36]. Müller glial cell production of vascular endothelial growth factor (VEGF), the major angiogenic factor, is known to be exacerbated in diabetes, where it contributes to vascular leakage and inflammation prior to neovascularization [36, 37].

In addition, under glycemic conditions, Müller cells also activated caspase-1 pathway, known to be a major source for IL-1β, which in turn, affects endothelial cell viability [38]. Besides IL-1β, Müller cells also produce a plethora of retinal neurovascular inflammation mediators, such as interleukin-6 (IL-6), IL-17, tumor necrosis factor-α (TNF-α), and chemokine ligand-2 (CCL-2), associated with vascular dysfunction and inflammation [39–43]. Moreover, aberrant Müller cell proliferation and release of pro-fibrotic mediators has been reported to play a pivotal role in the formation of fibroproliferative tissue [44], which represents an end stage in patients with proliferative diabetic retinopathy (PDR) and could cause retinal detachment [45]. Hence, Müller cells have been proposed as a source of myofibroblast in the epiretinal space where fibrovascular proliferation occurs [46], although the mechanism controlling Müller cell transdifferentiation in the context of diabetic retinopathy remains largely unknown [45].

In the brain, astrocytic alterations, on the other hand, have been profoundly described in other neurodegenerative diseases associated with diabetes, such as AD, where reactive astrogliosis is associated with amyloid plaques and Tau oligomers [47]. In the context of diabetes, however, the results are contradictory. While persistent astrocyte reactivity, identified by an enhanced glial fibrillary acidic protein (GFAP) expression and increased number of GFAP^+^ astrocytes, similar to that observed in AD, has been described in models of T1D and T2D in rats [48, 49], other authors reported attenuated GFAP expression in different areas of the brain of rats with T1D [50] or astrocytes exposed to hyperglycemia in vitro [51]. In the spinal cord, strong evidence points toward the relevance of reactive astrocytes in the development of diabetic peripheral neuropathy, mostly studied in animal models of T2D [52–55].

#### Crosstalk with neurons

The primary excitatory neurotransmitter responsible for learning, memory, and synaptic plasticity is glutamate, which in the retina also facilitates the transmission of visual signals from photoreceptors, bipolar cells, and ganglion cells. The clearance of glutamate from the extracellular space and its conversion into non-toxic glutamine is carried out by astrocytes in the CNS and Müller cells in the retina [34, 56] . Both, in DR and CD-AD, there are multiple lines of evidence of dysfunctional glutamate recycling leading to an excess of glutamate in the synapses, overstimulation of postsynaptic glutamate receptors, increased intracellular Ca2^+^ concentrations and subsequently, enhanced neuronal cell death [34, 56].

Glutamate transporter-1 (GLT-1) is the dominant glutamate transporter expressed by astrocytes in the brain and has been found to be significantly reduced or damaged in neurodegenerative diseases related with diabetes such as AD and cognitive impairment [57]. Despite the impairment in glutamate clearance [58], there are discrepancies about astrocyte GLT-1 expression in diabetic brains. While some groups reported no changes in the expression of GLT-1 and GLAST (glutamate/ aspartate transporter) despite an attenuated GFAP reactivity [50], others observed a marked reduction of GLT-1 [48]. Further studies aimed at elucidating alterations in glutamate transporters or recycling in diabetes and diabetes-associated CD are needed.

In the spinal cord, enhanced excitatory glutamatergic signaling has been associated with nociception in the context of diabetes [52, 59, 60]. The development of mechanical allodynia and hyperalgesia associated with T2D is usually linked to a decreased expression of GLT-1 particularly in the dorsal horn, and impaired extracellular glutamate recycling and accumulation [59].

Lastly, in the retina, Müller cells are essential to facilitate the use of glutamate for visual signal transmission by photoreceptors, and to do so, they express both, GLT-1 but mostly GLAST transporters, similarly to astrocytes, together with glutamine synthetase (GS), which in the retina is exclusively expressed in Müller cells [61]. Alterations in glutamate release and subsequent excitation of retinal ganglion cells have been observed already 4 weeks after the development of diabetes, being considered an early-stage diabetic symptom [62]. In experimental models of DR, an accumulation of glutamate occurs in the retina, either due to a significant decrease in glutamate transport via GLAST [62] or a decreased GS activity, necessary for neurotransmitter regeneration [63]. The expression of GLAST in diabetes has been reported to be significantly downregulated, leading to a higher amount of glutamate being transported to photoreceptor, bipolar, and ganglion cells [64]. Consistent with this, several reports showed an enhanced glutamate accumulation in experimental models of DR [65], with potentially toxic levels of glutamate detected in the vitreous humor of diabetic patients [66].

#### Glymphatic system

Fluid clearance is mediated by osmotic water transport coupled with the transport of osmolytes, mainly potassium, which alterations can also contribute to glutamate excitotoxicity. The main transporters involved in ion and water buffering are aquaporin-4 (AQP4) and inward rectifying K^+^ channel subtype 4.1 (Kir4.1), both located in Müller and astrocyte endfeet. Inwardly rectifying channels of the Kir family, mainly Kir4.1, play a critical role in spatial buffering of potassium ions that accumulate during neuronal activity, regulating the balance of water and glutamate uptake on astrocytes and Müller cells [67]. Both, Kir4.1 and AQP4, show an altered expression, location, and/or function in diabetic Müller retinal cells and brain and spinal cord astrocytes [55, 67, 68]. Astrocytes in hyperglycemic conditions and T2D mouse models show a significant reduction in the expression of Kir4.1 [64, 67], while Müller cells under diabetic stress conditions present an altered Kir4.1 location and activity [67, 69]. The redistribution of this channel in Müller cells has been recognized as the cause of reduced K^+^ conductance, that can result in an imbalance in K^+^ concentrations and disrupted K^+^ homeostasis and ultimately causing neuronal excitation and subsequent glutamate toxicity [34]. AQP4, on the other hand, shows an increased expression in astrocytes in hyperglycemic conditions [70], and although AQP4 role in DR is largely unknown, it has an increased expression in the vitreous of DR patients and in experimental models of DR, possibly as compensatory mechanism [71, 72]. This increase in AQP4 leads to impaired channel activity and glymphatic dysregulation [72, 73]. These alterations in Kir4.1 and/or AQP4 expression or activity in diabetic stress conditions are linked to a wide range of effects such as water and ion disruption homeostasis, Müller cell swelling, decrease glutamate recycling and gliosis, and even reduced Aβ clearance from the retina [34, 69].

In the brain, the glymphatic system contributes to Aβ and tau clearance, and the loss of polarity of AQP4 in the endfeet of astrocytes along with its upregulation in reactive astrocytes has been largely described in AD rodent models [74, 75]. Similarly, a dysfunctional glymphatic system has been also linked to cognitive impairment in T2D animal models [76, 77], possibly due to a downregulation of astrocytic Kir4.1 [68]. Likewise, spinal glymphatic system has been also found impaired in DPN due to reversed polarity of AQP4 in T2D rats [55, 78].

### Microglia

Tissue-resident macrophages and sentinels of the CNS, microglia represent 10–15% of human brain [79, 80]. Microglia adapt to different CNS microenvironments, expressing a range of markers, density, and morphology, thus displaying different functional features [81, 82]. Besides being considered the sentinels of the CNS, microglial functions also encompass neuronal network preservation and trophic support through synaptic pruning, neuronal debris clearance, synaptic control, myelination, angiogenesis, and blood barrier maintenance [80, 82–84]. The wide range of functions associated with microglia are facilitated by their capacity to migrate and perform constant CNS surveillance, to rapidly adapt and respond to CNS environmental changes together with their phagocytic activity [80, 82].

#### Gliosis and inflammation

Alterations in microglia have been observed not only in T2D animal models such as db/db mice, but also in diabetic patients, both with and without proliferative DR [81, 85]. Microglia response to sustained diabetic insults, include alterations in microglial migration and enhance secretion of pro-inflammatory and apoptotic microglial mediators in the retina, optic nerve, spinal cord, and brain [85–88].

The diabetic milieu, which comprises continuous exposure to hyperglycemia, oxidative stress, ischemia, and hypoxia, have been shown to alter microglial phenotypic states toward an ameboid shape, increased migration and proliferation, and release of a diverse repertoire of cytokines and chemokines in several diabetic animal models [81, 85]. In the diabetic retina, microglia have been described to migrate into the plexiform layer, where they acquire a hypertrophic morphology, surround neovascular areas and proliferate [88]. Ophthalmological examinations of patients with DR found hyper-reflective spots, corresponding with aggregates of activated microglia whose changes in number, migration, and phenotype in the retina correspond with the clinical progression of DR [86, 89]. These changes lead to a sustained microglial responsive phenotype that exacerbates DR pathology by perpetuating chronic neuroinflammation and neuronal damage [81, 90]. This is in part mediated by the increased microglial secretion of a plethora of pro-inflammatory cytokines and chemokines (e.g., TNF-α, IL-6, IL-1β, nitric oxide, CCL-2, and complement), which can accumulate in the vitreous, especially in those diabetic patients undergoing proliferative DR [81, 90, 91]. Besides their inflammatory role, microglia could also alter retinal vasculature in DR. Juxtavascular microglia, a type of microglia located close to the vasculature and previously described in the brain [92], has been associated with the phagocytic removal of dying pericytes and vascular smooth muscle cells in DR, which might have potential implications in the vasodegenerative pathology of DR[93].

In addition, bidirectional feedback between Müller cells and microglia has been described, especially after retinal injury such as DR, further sustaining gliosis and inflammation [40, 94]. In response to diabetic insults, Müller cells, possibly via CD40 receptor, elicit the secretion of chemokines CCL-2 and CCL-3 and increase the expression of VCAM-1 and ICAM-1 adhesion proteins, which guide the intraretinal microglial mobilization, further enhancing microglial inflammatory response [94, 95]. Besides, and in line with what has already been described for reactive neurotoxic astrocytes in AD [96], microglia strongly influence Müller cell morphology and function leading to retinal ganglion cell death [94].

In different experimental models of DPN secondary to both, T1D and T2D, spinal cord microglia show similar features to the ones described in DR, with an increased expression of microglia markers CD11b, enhanced phosphorylation in different kinases and hypertrophied microglia [97, 98]. These changes are more pronounced in the vicinity of the L4 segment in the dorsal horn [87], area that is innervated by myelinated fibers connecting the L4 dorsal root ganglia sensory neurons and the hind paw [99, 100]. This is consistent with several studies observing tactile allodynia in the hind paw of diabetic models and in the distal regions of diabetic patients such as feet and ankles. In line with what we have described for the retina, spinal cord microglia in diabetic models also show an increased production of common inflammatory cytokines and chemokines, namely TNF-α, IL-6, IL-1β, CCL-2, CCL-3, CCL-5, and CXCL-12, further contributing to DPN [99, 100].

In the brain, on the other hand, microglial alterations observed in diabetes are linked to those already extensively described in AD [7, 85, 101], including microglia increase in IBA-1 expression, proliferation, changes in morphology, and the presence of microglia that exhibit gene expression patterns associated with pro-inflammatory disease-associated microglia (DAM) [85, 101]. Moreover, T2D exacerbated the microglial pathology in different mouse models of AD, highlighting the potential overlapping pathological mechanisms between AD and diabetes. For example, in the mixed murine model of AD and T2D APP/PS1xdb/db mouse model, T2D significantly increased the number of microglia located in areas free of senile plaques as well as in the number of spontaneous hemorrhages [102]. On the other hand, in the APswe/Psi1dE9/TauP301L mouse model, when exposed to a typical western diet to induce diabetes, microglia response to β-amyloid and Tau pathology was reduced, with a decreased phagocytic uptake and amyloid plaque clearance, along with less microglia located also in the vicinity of β-amyloid plaques in obese individuals with T2D than in those without T2D [103].

These observations have been validated through mechanistic studies in which primary microglia have been exposed to hyperglycemic conditions, which enhanced their proliferation and the production of pro-inflammatory factors such as TNF-α, CCL-2, oxygen radicals, stress proteins (e.g., HSP80), reactive oxygen species (ROS), heme oxygenase 1 (HO-1), and inducible nitric oxide synthase (iNOS), further maintaining pro-inflammatory microglial phenotype [81, 104, 105]. Interestingly, microglial cells that were under hyperglycemia and were then returned into normoglycemic conditions showed increased metabolic stress, bcl-2 and caspase-3-mediated apoptosis and autophagy, potentially further contributing to neurovascular complications [105].

Therefore, the continuous hyperglycemic insult in diabetes greatly impact microglia phenotype and function across the different regions of the CNS (optic nerve, retina, brain, and spinal cord), exacerbating microglial release of pro-inflammatory mediators and morphological shift to ameboid, creating a feedback loop that sustains chronic inflammation, and thus neurodegeneration.

### Oligodendrocyte lineage cells

Oligodendrocyte lineage cells encompass two groups of cells, on the one hand oligodendrocyte progenitor cells (OPCs) which are responsible for giving raise to oligodendrocytes in development and in response to injury, and on the other hand oligodendrocytes, the cells responsible for forming myelin in the CNS [106]. Myelin is an insulating lipid rich layer that surrounds axons, providing physical protection, trophic support and allowing the fast saltatory conduction of action potentials [107]. Even though myelin alterations in the CNS were initially associated exclusively with primary demyelinating diseases, such as multiple sclerosis or leukodystrophies, it is now widely accepted that myelin alterations are present in a wide range of neurological disorders including autism spectrum disorders, Parkinson’s disease, or AD [108]. As we mentioned, even if diabetes is not initially considered a neurodegenerative disease, it shares many CNS neurodegenerative hallmarks with AD, and myelin alterations are not an exception.

Loss of myelin sheaths has already been extensively described both, in diabetic CNS and PNS, around the 1960s [109, 110], with white matter hyperintensities observed in MRI of diabetic patients [111]. Already in the 1980s, it was established that the whole brain and especially brain myelin undergo excessive glycosylation in diabetes, with 3.8 more glycosylation than normal brain myelin, which may contribute to the functional abnormalities of myelinated neurons observed in diabetes [112]. However, the specific changes underlying myelin loss in the diabetic CNS are only starting to be elucidated. It has now been established that myelin alterations are present early, prior to neurodegeneration, both, first in the PNS and then in more advanced diabetic stages, in the CNS. Lipid profile analysis of the cortex of STZ-treated diabetic rats revealed that diabetes alters myelin composition in the CNS impacting phosphatidylcholine, phosphatidylethanolamine, plasmalogen, cholesterol, and polyunsaturated fatty acid levels, which are significantly decreased in the cerebral cortex myelin. Besides changes in myelin lipid composition, alterations in myelin basic protein (MBP), the most abundant myelin protein but not proteolipid protein (PLP) were also observed in STZ-treated rats. Surprisingly, treatment with dihydroxyprogesterone every other day for a month reversed all these changes, restoring myelin composition to the levels of healthy control rats [113]. Work performed by a different research group addressed spatiotemporal lipidomic changes in the CNS of db/db mice at 1, 2, and 4 months of age, further validating and expanding the previously described alterations in CNS myelin lipid composition. Sulfatide and cerebrosides were found significantly decreased in the spinal cord but not the brainstem of 1-month-old db/db mice and even further decreased at 2 and 4 months old. At 2 months old, not only sulfatides and cerebrosides, but also plasmalogens were reduced in the CNS, both in the spinal cord and the brainstem, while phosphatidylcholine levels were not altered. In vivo electrophysiological studies showed that myelin alterations in the CNS preceded neuronal functional changes, as electrophysiological changes were only detected in db/db mice at 4 months. Myelin ultrastructural analysis revealed no changes at 4 months between control and db/db mice in the CNS, suggesting that lipid changes occur prior to late-stage myelin loss in the CNS. These results were replicated in other diabetic models including high-fat diet model and STZ model [23].

Myelin loss is generally rapidly restored through a process known as remyelination, a regenerative process by which OPCs respond to myelin damage differentiating into myelinating oligodendrocytes and restabilizing myelin around denuded CNS axons [106]. However, this response appears to be hampered in diabetic models, which show an impaired oligodendrogenesis, aggravating symptoms and neuronal damage observed in other injuries such as stroke. T2D multiplies the risk of developing stroke fourfold by increasing neuronal tissue loss, white matter damage, and limiting oligodendrogenesis. OPC proliferation and the generation of new myelinating oligodendrocytes was significantly impaired in db/db mice upon middle cerebral artery occlusion, with significantly lower NG2^+^BrdU^+^ cells in the cortex. This translates into a decrease in the amount of MBP protein present 35 days upon stroke in db/db mice [114]. These results were also observed in another model of stroke, bilateral common carotid artery stenosis, in db/db mice. Diabetic mice also showed a decrease in the number of GSTpi^+^ oligodendrocytes 4 and 8 weeks after ischemia, as well as in the number of PDGFRα^+^ OPCs and proliferating OPCs. In addition, a decreased survival of proliferating OPCs upon ischemic stroke was also detected in db/db mice compared to heterozygous db/ + controls. Thus, diabetic mice exhibit a more severe white matter injury and a poorer recovery upon ischemic stroke, suggesting that diabetes may impair myelin regenerative capacity [115]. Similarly, STZ-treated mice show less exploratory behavior and more anxiety 4–8 weeks after treatment started, which is associated with a decrease in the number of OPC and MBP^+^ oligodendrocytes. This result is further supported by in vitro experiments, in which a hyperglycemic environment impairs OPC survival and migration, two key steps in CNS myelin regeneration [116]. These results are also replicated in the optic nerves of STZ-treated mice, which show a decrease in the number of oligodendrocytes and a decreased expression of PLP and MAG myelin proteins and nodal proteins. Electron microscopy analysis confirmed the decreased number of myelinated axons and nodes of Ranvier, corroborating myelin alterations in diabetic optic nerve. Treatment with Clemastine, a muscarinic receptor agonist shown to be able to restore remyelination, restored oligodendrocyte number and promoted optic nerve functional recovery in STZ-diabetic mice [117]. In addition, Metformin, a commonly used anti-diabetic drug, has recently been shown to have potent pro-remyelinating effects, potentially alleviating some of the cognitive symptoms observed in diabetic patients through this mechanism [118].

Therefore, systemic alterations underlying diabetes affect both, myelin maintenance and regeneration in the CNS. Drugs known to restore myelin regeneration in the CNS such as Clemastine or Metformin, are potentially capable of reversing some of the diabetic-induced neurological alterations at least partially through changes in myelin, suggesting that pro-myelinating drugs may open a new window of opportunity to prevent diabetes-associated neurodegeneration.

## Peripheral nervous system glia

### Schwann cells

SCs are the most abundant glial cells of the PNS and ensheath either myelinating or unmyelinating peripheral nerve axons [119, 120]. SCs are essential in maintaining conduction of electrical impulses along PNS axons, supporting nerve development and regeneration, and providing peripheral nerve axons metabolic and neurotrophic support [121]. DPN is one of the most common complications of diabetes, affecting more than half of the people suffering from diabetes during the course of the disease. Neuronal damage leading to DPN is associated with diabetes-induced changes in SCs that jeopardize the production and release of neuronal support factors and lead to the accumulation of neurotoxic and pro-inflammatory factors, such as (TNF)-α, interleukin (IL)-1α, IL-1β, MCP-1, CCL2, LIF, and CXCL2, contributing to the endothelial dysfunction, axonal degeneration, and neuronal damage underlying DPN [119, 122, 123]. Even if the nature of the primary lesion in DPN (demyelination or axonal loss) is still controversial, it is now well established that SCs play a key role in DPN pathogenesis.

#### Innervation

Autonomic dysfunction associated with altered innervation is common in DPN and affects several important functions. DPN is characterized by a progressive, distal-to-proximal degeneration of peripheral nerves, with unmyelinated small caliber nerve fibers being impacted first, followed by large-myelinated fibers [124]. Experiments assessing footpad autonomic innervation and sweating in db/db mice unveiled that unmyelinated fibers innervating sweat glands were more prominently affected than their unmyelinated epidermal sensory counterparts in the early diabetic stages [125]. Besides, db/db mice display progressive sensory loss and electrophysiological impairment in the early to-mid phases of the disease partially, at least, due to a decrease in intraepidermal nerve fiber density, and thus sensory loss. This sensory loss is highly associated with SC apoptosis and T cell infiltration in the sciatic nerve, which perpetuate inflammation and neuronal loss [126]. In addition, recent studies have also shown that DPN can affect adipose tissue innervation. Subcutaneous white adipose tissue (scWAT) contains both, myelinated and unmyelinated nerves, as well as SCs linked to synaptic vesicle-containing nerve terminals, suggesting that SC may be involved in regulating tissue nerve plasticity. However, in a type 2 diabetes model based on BTBR which carries a homozygous spontaneous and recessive mutation (Lep^ob^) in the leptin gene (ob/ob mice), the adipose tissue develops small fiber demyelinating neuropathy with changes in SC marker gene expression, like what has been described in diabetic human adipose tissue. Thus, SC regulation of tissue nerve plasticity may also become altered as a consequence of diabetes [127].

##### Impaired neurotrophic support

During SC maturation and alignment, a variety of neurotrophic factors and adhesion molecules, such as nerve growth factor (NGF), brain-derived neurotrophic factor (BDNF), and neurotrophins (NT-3, NT-4 or NT-5), are released, which orchestrate neuronal sprouting and targeting [128, 129]. Several studies have shown that diabetes impacts SC capacity to produce neurotrophic factors, limiting their capacity to support axonal growth and thus, contributing to DPN development and progression. For example, SCs show a decreased production of BDNF in the sciatic nerve of STZ-treated diabetic animals, similarly to SC ciliary neurotrophic factor-like activity, which is also reduced in SCs upon STZ treatment. This decrease in BDNF secretion is associated with the increased systemic glucose in diabetes, as SCs in culture under hyperglycemic conditions show reduced BDNF levels [130]. Similarly, SCs in hyperglycemic conditions show an impaired production and secretion of both, NGF and NT-3, which are essential for peripheral nervous system regeneration. This decline in NGF and NT-3 translates into an impaired neurite outgrowth of dorsal root ganglion and a poor association between neurons and SCs in SC-dorsal root ganglion co-cultures in diabetic conditions [131]. Moreover, in a situation of peripheral nerve injury, SCs have the capacity to reprogram and de-differentiate into repair SC phenotype that provides support and accelerates repair [132]. However, in db/db mice, skin wound healing was delayed and SCs failed to activate their repair program in a timely manner due to functional impairments in cell de-differentiation, cell-cycle re-entry and cell migration, problems associated with hyperglycemia as shown by in vitro experiments [133].

#### Inflammation

Recently, SCs have been recognized as immune-competent cells, exhibiting immune functions like the non-myelinating glia of the CNS [134, 135]. SCs can express major histocompatibility complex II molecules as well as adhesion molecules, several toll-like and inflammatory receptors and produce several cytokines and chemokines. These processes become exacerbated in the inflammatory conditions present in DPN. Under high glucose and oxidative stress conditions, like those observed in diabetes, SCs express increased levels of nuclear factor-κβ (NF-κβ), Toll-like receptors (e.g., TLR4) and pro-inflammatory cytokines and chemokines such as TNF-α, IL-6, IL-1β, CXCL9, CXCL10, and CXCL11 [120, 136–138]. These inflammatory cytokines can directly sensitize Aδ and C-fibers or contribute to the activation and recruitment into DPN tissues of other immune cells such as macrophages and CD8^+^Tcells, which in turn can trigger cytotoxicity by activating apoptosis toward SCs, thus perpetuating injury [136]. In addition, SCs p75 neurotrophin receptor (p75^NTR^), which is significantly downregulated in diabetes, regulates the neuroinflammatory landscape and promotes phagolysosomal remodeling in high-fat-diet-induced DPN. Therefore, the decreased expression of p75^NTR^ in diabetic-like SCs leads to a significant upregulation of genes associated with inflammatory and immune functions such as *Il7r, Serpina3n* or *Cxcl13* in SCs, suggesting that SCs have a primary contribution to DPN, as this pro-inflammatory phenotype may contribute to peripheral nerve degeneration [139].

#### Myelin maintenance

As mentioned previously, myelin alterations associated with diabetes were already described in the PNS in the 1960s [109, 110]. Electron microscopy images of the sural nerve of diabetic patients with or without neuropathy showed that even if fibers had similar diameter, peripheral nerve myelin sheaths of patients with DPN not only had fewer lamellae but also showed segmental demyelination and remyelination, suggesting a primary deficit in SCs [109, 140, 141]. To uncover the specific alterations underlying myelin changes in DPN, several groups have studied peripheral nerve myelin composition. An initial lipidomic analysis revealed that myelin of STZ-treated rats sciatic nerve showed a decrease in phospholipids, fatty acid, and cholesterol content. In line with this, they also observed a reduced expression of the genes associated with fatty acid biosynthetic pathway as well as a decreased expression of sterol regulatory element binding factor-1c (SREBF-1c), a key lipogenic factor. In addition, myelin protein P0, which is one of the major components of sciatic nerve myelin, was also decreased in STZ-treated rats. Electron microscopy analysis showed that STZ-treated rats had an increased number of abnormal fibers and myelin infoldings. STZ-treatment-associated PNS phenotype was surprisingly reversed by the activation of the nuclear receptor liver X receptor (LXR), a gene upstream SREBF-1c and a major regulator of fatty acid biosynthesis [142]. More recent spatiotemporal lipidomic analysis of db/db mice, STZ-treated mice, and mice exposed to high-fat diet validated this initial diabetic PNS myelin lipid profile and showed an altered lipid composition already in 1-month-old db/db mice. Lipidomic results revealed a significant decrease in total sulfatide, cerebrosides, and plasmalogen levels in PNS myelin, while phosphorylcholine was not altered. These changes were further exacerbated with age in the PNS. Electrophysiological studies to determine the conduction velocities of peripheral nerves in these mice show no alterations at 2 months of age, indicating that myelin alterations proceed axonal loss in the PNS. Electron microscopy analysis of myelin in 4-month-old db/db confirmed a significant decrease in average PNS myelin thickness, in line with the observed lipidomic changes, while axon caliber was not affected. These alterations in lipid content also translated into changes in both, myelin proteins and mitochondrial function in the PNS, as indicated by an increase in MBP at 1 month of age that was rapidly reverted into an MBP loss by 2 and 4 months of age, and significant effect in oxygen flux in sciatic nerve, indicating that global respiratory capacity was significantly impaired in *db/db* mice [23]. Myelin thinning and the presence of patches of demyelination and remyelination in the PNS suggest that demyelination is a pathological feature of DPN, even though the mechanisms behind DPN-linked demyelination remain unclear. This PNS demyelination could at least in part be caused by SC-mediated myelin degradation. A recent publication has shown a key role for mixed lineage kinase domain-like protein (MLKL), which participates in regulating necroptosis and is induced in SCs in STZ-induced diabetic model. Moreover, MLKL upregulation and subsequent phosphorylation in the serine 441 is responsible for SC-mediated demyelination and subsequent decrease in nerve conduction velocity, which was prevented either by SCs-specific MLKL knockout or by inhibiting MLKL pharmacologically [143]. An alternative or additional mechanism that may potentially contribute to PNS demyelination in DPN involves the formation of advanced glycation end products (AGEs). The formation of AGE has been liked to PNS myelin in diabetic neuropathy. PNS myelin modified by AGEs is more susceptible to macrophage-mediated phagocytosis, and thus facilitates myelin loss. Altogether, AGEs-modified PNS myelin together with changes in major axonal cytoskeletal proteins derived from AGEs-based modifications translate into impaired axonal transport and subsequent nerve degeneration [138].

Therefore, SCs play a key role in several aspects of DPN including PNS nerve innervation, myelination, and demyelination and the expression of inflammatory molecules that perpetuate peripheral chronic inflammation. Even it is yet to be determined whether the first event triggering DPN is axonal loss or demyelination, there is mounting evidence pointing toward a role for SCs in DPN pathology.

### Satellite glial cells

SGCs are located surrounding neuronal soma in the sensory and autonomic PSN ganglia, establishing a narrow 20 nm gap between the neuron and SGCs, which allows a tight control of the neuronal extracellular space and a bidirectional communication between neurons and SGCs [144]. It is now well established that SGCs express neurotransmitter receptors, transporters, and ion channels, been capable of monitoring neuronal activity and responding to neuronal stress by proliferation, upregulation of GFAP, connexin 43 and P2 receptors [145]. Even though their biology remains largely unknown, their close communication with the perineuronal environment makes them an attractive target to treat peripheral neuropathies.

SGC to neuron signaling is regulated by the P2X7 receptor, which activation leads to the release of TNF-α and ATP by SGCS and subsequently drives neuronal hyperexcitability [25]. In STZ-diabetic models, SGCs increase GFAP expression through the upregulation of P2X7. Inhibiting upregulation of P2X7 receptors in SGCs reduced SGCs response and subsequent TNF-α release, decreasing dorsal root ganglia (DRG) hyperexcitability, and thus limiting thermal and mechanical hyperalgesia in diabetic rats [25, 146–148]. Besides, hyperglycemia has been recently shown to induce an increase expression of pro-inflammatory lipocalin-2 (LCN2) in DRG SGCs, potentiating neuroinflammation and neurotoxicity via LCN2-pyruvate dehydrogenase kinase isoform 2 (PDK2)-lactic acid pathway contributing to the progression of DPN [146]. Lastly, a recent study pointed toward a potential role of septin-9 (SEPT9), an upstream regulation of a N-methyl-D-aspartate receptor subunit NR2B on SGCs as a mechanism of mechanical nociception in PDN [149].

Hence, despite not being fully characterized yet, SGCs appear to contribute to DPN by modulating neuronal excitability and therefore altering the response to thermal and mechanical stimuli in DPN. Due to their proximity to DRG neurons and their close bidirectional communication, SGCs appear as a promising future target to modulate and limit DPN progression.

## Enteric nervous system glia

### Enteric glial cells

Diabetes can lead to various complications affecting multiple organs, and the gastrointestinal (GI) tract is no exception. Diabetic patients often suffer from GI motility disorders that result in symptoms such as nausea, vomiting, bloating, and constipation. Although the underlying mechanisms of these disorders are not yet fully understood, recent studies have highlighted the potential role of enteric glial cells (EGCs) in diabetes-associated GI pathophysiology [150].

EGCs are a population of non-myelinating glial cells and the most numerous glial cell type in the enteric nervous system (ENS), which is a complex network of neurons and glia that controls GI function. EGCs play crucial roles in maintaining ENS function such as supporting the integrity and function of enteric neurons and regulating GI motility, inflammation, and epithelial barrier function. EGCs develop from neural crest precursors during embryonic days 9 to 13.5 in mice, and cells expressing markers of terminally differentiated glia such as S100β and GFAP are present by E14.5–16 [151, 152]. Mature EGCs are similar in morphology to astrocytes and express similar molecular markers, such as Aldh1L1. They also express SCs or oligodendrocyte markers such as Sox10 and Plp1 [153]. In addition, EGCs share the capacity of astrocytes to regulate tight-junction integrity and cellular interactions comparable with those maintaining the blood–brain barrier, creating the proper microenvironment for enteric neurons [154].

Several studies have investigated the potential involvement of EGCs in the pathophysiology of GI complications in diabetes. Autonomic neuropathy caused by diabetes mellitus is related to quantitative and morphometric changes in the enteric neurons in various GI segments, associated with the reduced levels of glutathione observed in diabetic patients [155, 156]. In addition, neurons depend directly on glial cells for glutathione synthesis, while glial cells directly promote neuronal protection by increasing the intracellular content of total glutathione and protecting enteric neurons against oxidative stress [157, 158]. Furthermore, glial preservation may be attributable to the resilience of the glial cell population and a defense mechanism exerted by glia in an attempt to promote the maintenance of neurons after the development of peripheral diabetic neuropathy [155]. In addition, EGCs secrete neurotrophic factors, such as glial-cell-derived neurotrophic factor (GDNF), NGF, and transforming growth factor-beta (TGF-β), which contribute to the maintenance of endothelial integrity and vasodilation. EGC-mediated GDNF secretion rescues hyperglycemia-mediated enteric neuronal loss through the activation of the PI3K/Akt pathway [159, 160]. However, in diabetes, EGCs can also present a reduction in the expression of neurotrophic factors or neurotrophins responsible for promoting neuronal survival and maintenance, thus contributing to the development of the disease [155, 160]. Besides changes in neurotrophic factors, EGCs also present alterations in GFAP expression in diabetes. GFAP expression changes in EGCs could be the consequence of unviable extracellular conditions such as hyperosmolarity, low nutrient availability or increased oxidative stress [160]. These findings suggest that there are complex interactions between enteric neurons and glial cells, which play an important role in the pathogenesis of diabetic enteric neuropathy.

In summary, EGCs play critical roles in the regulation of GI function and may contribute to the pathophysiology of GI complications in diabetes. Understanding the mechanisms underlying EGC dysfunction in diabetes and developing new therapies targeting EGCs may provide promising avenues for the treatment of GI motility disorders in diabetic patients.

## Concluding remarks

**Fig. 2:**
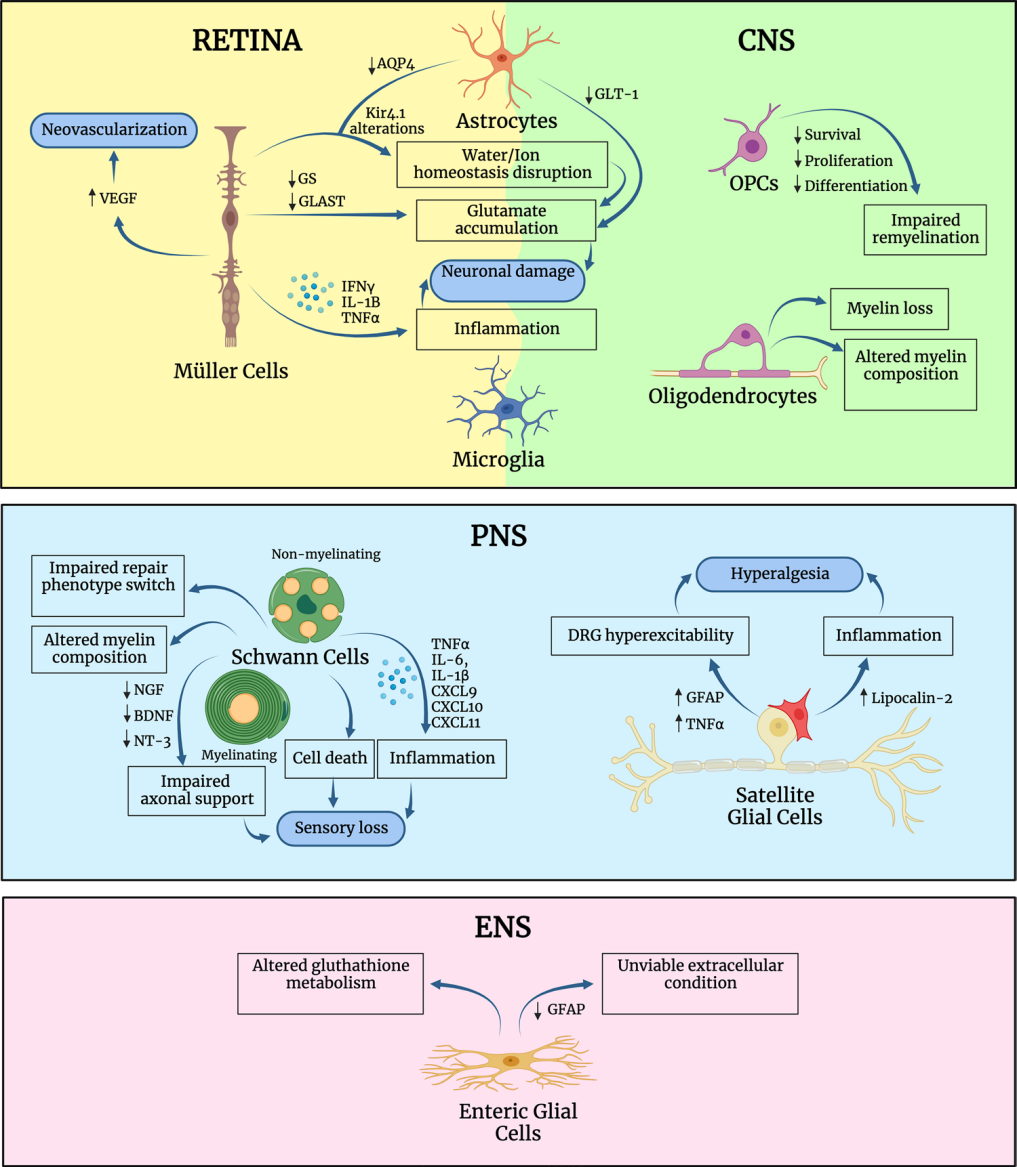
Summary of the glial cell alterations described in the most common neural diabetic complications. Glial cells show altered functions in the retina, as well as the peripheral and enteric nervous system, where they share common and unique alterations in response to diabetes. This figure was created with BioRender.com. *AQP4* aquaporin-4, *BDNF* brain-derived growth factor, *CNS* central nervous system, *CXCL* C-X-C motif chemokine ligand, *DRG* dorsal root ganglia, *ENS* enteric nervous system, *GFAP* glial fibrillary acidic protein, *GLAST* glutamate aspartate transporter, *GLT-1* glutamate transporter-1, *GS* glutamine synthase, *IFN* interferon, *IL*- interleukin, *NGF* nerve growth factor, *NT* neurotrophin, *PNS* peripheral nervous system, *TNF* tumor necrosis factor, *VEGF* vascular endothelial growth factor

This review summarizes glial cell alterations observed in the three most common and disabling neural diabetic complications, namely DR, DPN, and cognitive decline. Glial cells are known to have key roles in neuronal support and maintenance in the CNS, the PNS, and the ENS, which are significantly altered in diabetes (Fig. 2). Thus, understanding the mechanisms by which glial cells contribute to the pathogenesis and neurological progression of diabetes will open new avenues to develop therapeutic approaches aiming at preventing neurodegeneration, both in the CNS and PNS. Limiting neurodegeneration will restrain DR, DPN, and cognitive decline progression, thus decreasing the main health and economic burden associated with diabetes, and thus restricting the impact of diabetes in the society.

## Data Availability

Not applicable.

## Abbreviations

Aβ: Amyloid β
AD: Alzheimer’s disease
AGEs: Advance glycation end products
APP/PS1: APPswe/PS1ΔE9, double transgenic mice expressing a chimeric mouse/human amyloid precursor protein (Mo/HuAPP695swe) and a mutant human presenilin 1 (PS1-dE9) Alzheimer’s disease mouse model
AQP4: Aquaporin-4
ATP: Adenosine tri-phosphate
BBB: Blood–brain barrier
BDNF: Brain-derived neurotrophic factor
BrDU: Bromodeoxyuridine
BRB: Blood–retinal barrier
BTBR ob/ob: *ob/ob* Mice on a BTBR background (BTBR.Cg-*Lep*.^*ob*^/WiscJ)
CCL: Chemokine ligand
CXCL: C-X-C motif chemokine ligand
CD: Cognitive decline
CD11b: Macrophage-1-antigen, also known as OX-42
CD-AD: Cognitive decline and Alzheimer’s disease
CNS: Central nervous system
DAM: Disease-associated microglia
db/db: Type 2 diabetes mouse model (Lepr^db^)
DPN: Diabetic peripheral neuropathy
DR: Diabetic retinopathy
DRG: Dorsal root ganglion
EGC: Enteric glial cells
ENS: Enteric nervous system
GDNF: Glial-cell-derived neurotrophic factor
GFAP: Glial fibrillary acidic protein
GI: Gastrointestinal tract
GLAST: Glutamate aspartate transporter
GLT: Glutamate transporter
GS: Glutamine synthetase
ICAM-1: Intercellular adhesion molecule
IL: Interleukin
iNOS: Inducible nitric oxide synthase
Kir4.1: Inward rectifying K^+^ channel subtype 4.1
LCN2: Lipocalin-2
MBP: Myelin basic protein
MLKL: Mixed lineage kinase domain-like protein
NG2: Neural/glial antigen 2
NGF: Nerve growth factor
NT: Neurotrophin
NVU: Neurovascular unit
OPC: Oligodendrocyte progenitor cells
PDK2: Pyruvate dehydrogenase kinase isoform 2
PDR: Proliferative diabetic retinopathy
PDGFR: Platelet-derived growth factor receptor
PLP: Proteolipid protein
PNS: Peripheral nervous system
ROS: Reactive oxidative species
S100β: S100 calcium binding protein β
SC: Schwann cell
SCs: Schwann cells
scWAT: Subcutaneous white adipose tissue
Sept9: Septin 9
SGC: Satellite glial cells
SREBF-1c: Sterol regulatory element binding factor 1c
STZ: Streptozotocin
T1D: Type 1 diabetes
T2D: Type 2 diabetes
TGF: Transforming growth factor
TLR: Toll-like receptors
TNF: Tumour necrosis factor
VCAM-1: Vascular adhesion molecule
VEGF: Vascular endothelial growth factor
